# Supplementary material for: Causal Relationship between Aging and Anorexia Nervosa: A White-Matter-Microstructure-Mediated Mendelian Randomization Analysis
Source: Biomedicines. 2024 Aug 16;12(8):1874. doi: 10.3390/biomedicines12081874 (PMC11351342; doi:10.3390/biomedicines12081874)
Supplement: Supplementary file 1 [file biomedicines-12-01874-s001.zip › Supplementary Figure/Figure S8/Leaveoneout plot of MR analysis for TL to ubm-b-2076.pdf]

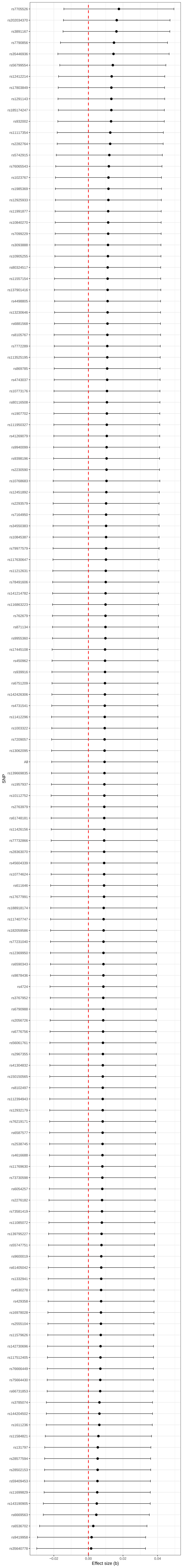

rs7705526
rs202034370
rs3891167
rs7790856
rs35446936
rs56799554
rs12412214
rs17803849
rs1291143
rs185174247
rs932002
rs11117354
rs2282764
rs5742915
rs76065543
rs1023767
rs1985369
rs12925933
rs11991877
rs10840270
rs7099229
rs3093888
rs10905255
rs80324517
rs11557154
rs137901416
rs4498805
rs13230646
rs6881568
rs8105767
rs7772289
rs113525195
rs869785
rs4743037
rs10773176
rs80116508
rs1907702
rs111850327
rs41269079
rs9940099
rs9398196
rs2230590
rs10768683
rs12451892
rs2293579
rs7164950
rs34550383
rs10845387
rs79977579
rs117630647
rs11212631
rs78491606
rs141214782
rs116863223
rs762679
rs871134
rs9955360
rs17445108
rs450962
rs939916
rs6751209
rs142426306
rs4731541
rs11412296
rs1003322
rs7209057
rs13062095
All
rs139669835
rs1957937
rs10112752
rs2763979
rs61748181
rs11426156
rs77732866
rs28363070
rs45604339
rs10774624
rs611646
rs17677991
rs188918174
rs117407747
rs182059586
rs77231040
rs12369950
rs6590343
rs9878436
rs4724
rs3767952
rs6790988
rs2056726
rs6776756
rs56061761
rs2967355
rs41304832
rs150150565
rs8102497
rs112394943
rs12932179
rs76219171
rs6587577
rs2538745
rs4616688
rs11769630
rs73730598
rs6054257
rs2276182
rs73581419
rs11085072
rs139795227
rs55747751
rs9600019
rs61405042
rs1332941
rs4530278
rs429358
rs16978028
rs2555104
rs11579626
rs142730696
rs117512405
rs76666449
rs75664430
rs66731853
rs3785074
rs144204502
rs1611236
rs11584821
rs131797
rs28577594
rs28502153
rs59409453
rs11690829
rs143190905
rs6669563
rs6536702
rs9419958
rs35640778
SNP
-0.02
0.00
0.02
0.04
Effect size (b)
